# Graph attention networks for predicting drug-gene association of glucocorticoid in oral squamous cell carcinoma: A comparison with GraphSAGE

**DOI:** 10.1371/journal.pone.0327619

**Published:** 2025-07-03

**Authors:** Monal Yuwanati, Santhanamari Thiyagarajan, Kranti Kiran Reddy Ealla, Yash Jain, Pradeep Kumar Yadalam, Senthil Murugan Mullainathan, Anima Nanda, Samir Sahoo, Daniel Ejim Uti

**Affiliations:** 1 Department of Oral and Maxillofacial Pathology, Saveetha Dental College and Hospitals, Saveetha Institute of Medical and Technical Sciences, Saveetha University, Chennai, India; 2 Department of Medical Laboratory Technology, Faculty of Applied Medical Sciences, Northern Border University, Arar, Kingdom of Saudi Arabia; 3 Department of Oral and Maxillofacial Pathology, Malla Reddy Institute of Dental Sciences, Malla Reddy Vishwavidyapeeth, Hyderabad, India; 4 Department of Dentistry, University of California Los Angeles, Los Angeles, California, United States of America; 5 Department of Periodontics, Saveetha Dental College and Hospitals, Saveetha Institute of Medical and Technical Sciences, Saveetha University, Chennai, TamilNadu, India; 6 Department of Oral and Maxillofacial Surgery, Saveetha Dental College and Hospitals, Saveetha Institute of Medical and Technical Sciences, Saveetha University, Chennai, India; 7 Department of Biomedical, Sathyabama Institute of Science and Technology, Chennai, Tamil Nadu, India; 8 Department of General Medicine IMS and SUM Hospital, Siksha ‘O’ Anusandhan (Deemed to be University), Bhubaneswar, Odisha, India; 9 Department of Biochemistry/Research and Publications, Kampala International University, Kampala, Uganda; 10 Department of Biochemistry, Faculty of Basic Medical Sciences, College of Medicine, Federal University of Health Sciences, Otukpo, Benue State, Nigeria; The First Hospital of Jilin University, CHINA

## Abstract

**Background:**

The present study evaluates the effectiveness of Graph Attention Networks (GAT) and GraphSAGE in predicting drug-gene interactions for glucocorticoids in oral squamous cell carcinoma, thereby aiding in developing better treatment strategies.

**Materials and methods:**

We utilized a curated dataset containing known drug-gene interactions and corresponding molecular profiles. Both GAT and GraphSAGE were implemented to model the biological networks of drug-gene relationships. Experiments were conducted to evaluate each model’s performance using accuracy, precision, recall, and F1-score metrics.

**Results:**

The network analysis details 174 nodes and 409 edges with a sparse structure, moderate connectivity, and low clustering, indicating a diverse node connection. The analysis confirms a fully connected network with efficient computation time. In comparing models, GraphSAGE outperforms GAT with higher accuracy (0.949 vs. 0.947), better macro-averaged F1 score (0.275 vs. 0.195), and higher AUC-ROC (0.780 vs. 0.514), suggesting stronger class-distinction capabilities. Both models achieve high accuracy, but GraphSAGE’s superior scores in F1 and AUC-ROC indicate a more effective balance in precision and recall. The results demonstrated that both GAT and GraphSAGE effectively predicted drug-gene associations. However, GAT outperformed GraphSAGE, achieving higher accuracy and F1 scores in identifying relevant glucocorticoid interactions in the context of OSCC.

**Conclusion:**

Our findings highlight the efficacy of advanced graph-based methodologies in elucidating drug interactions in OSCC. GAT, in particular, shows promise for accurately predicting drug-gene associations, which may facilitate personalized therapeutic approaches. Future research will focus on enhancing these models and exploring additional drug compounds to understand their applicability in OSCC treatment.

## Introduction

Oral squamous cell carcinoma (OSCC) is a prevalent malignancy worldwide, with a significant prevalence in regions with high tobacco and alcohol consumption [1]. OSCC is responsible for nearly 3% of all cancers, with rates that are considerably higher in regions such as Southeast Asia, according to global cancer statistics [2]. The disease is associated with a high mortality rate, particularly owing to late-stage diagnosis, resulting in an overall 5-year survival rate of approximately 50% [3]. Additionally, OSCC is associated with significant morbidity, which adversely affects patients’ quality of life by complicating their social interactions, speech, and nutrition [4–6].

The primary and traditional therapeutic modalities for OSCC include surgical resection, radiation therapy, and chemotherapy. Surgery is often the first-line intervention; nonetheless, it can lead to considerable morbidity and functional deficits [7]. Adverse effects, such as mucositis and xerostomia, detrimentally affect patient quality of life and are similarly observed in radiation therapy [8]. Chemotherapy is associated with notable side effects and demonstrates limited effectiveness in advanced stages of OSCC [9]. Moreover, the heterogeneity of OSCC poses a substantial challenge in treatment response, highlighting a critical gap in our understanding of personalized medicine strategies [10].

Recent advancements have facilitated the exploration of targeted therapeutic strategies focusing on specific molecular pathways implicated in OSCC [9]. Although targeted therapies, including EGFR inhibitors, have demonstrated potential, their clinical utility is limited by developing resistance mechanisms and heterogeneity in patient response [11]. Consequently, there is a pressing need to develop more productive and personalized therapeutic approaches to effectively address the underlying biological and genetic variability inherent in OSCC.

The glucocorticoid receptor (GR), encoded by the Nuclear Receptor Subfamily 3 Group C (NR3C) gene, regulates cellular metabolism and immune function. Glucocorticoids, acting via the glucocorticoid receptor, exert multifaceted effects on neoplastic cells, particularly in OSCC, influencing inflammatory pathways, cell proliferation, and apoptotic mechanisms [12]. Despite their antiproliferative properties, prolonged glucocorticoid exposure has been associated with tumor progression and metastasis, implicating a dualistic role that complicates their therapeutic application [13]. This duality necessitates comprehensive research into their function in OSCC pathogenesis and progression.

Considering the role of the GR gene in diverse oncogenic processes, targeting it emerges as a viable strategy for OSCC therapy. This approach holds the potential for developing innovative therapeutic interventions capable of inhibiting or modulating GR signaling pathways to augment the efficacy of existing treatments [14]. Elucidating the molecular mechanisms by which the GR gene affects tumor behavior is crucial for identifying biomarkers pertinent to prognosis and therapeutic response, facilitating personalized treatment strategies for OSCC. The GR gene is a promising focal point for future research endeavors to enhance clinical outcomes in OSCC. Further, exploring GR gene-targeted therapies in conjunction may reveal novel methodologies to rectify the substantial deficiencies in current therapeutic approaches.

Predicting drug and gene associations in GR is crucial for personalized medicine, improved treatment outcomes, biomarker development, and understanding resistance mechanisms [15,16]. Understanding how GR interacts with specific drugs can lead to more tailored treatment plans, reducing side effects and improving response rates. Identifying gene expressions associated with GR pathways can help develop biomarkers for early detection, treatment response, and prognosis. Researchers use data collection, bioinformatics, laboratory experiments, and clinical trials to predict OSCC GR interactions, improving patient outcomes and informed therapeutic decisions.

Graph-based network analysis is crucial in machine learning and deep learning for representing complex relationships, improving interpretability, and providing computational efficiency [17]. It allows attention mechanisms to focus on relationships across different levels of granularity, enhancing global context understanding and dynamic attention. Graph representations reduce computational burden, facilitate parallel processing, and promote transparency in model decisions. One previous study showed that graph attention networks predict therapeutic gene-disease links using data from biomedical sources. Graph Attention Networks (GAT) improves specificity and accuracy in clinical trial outcomes, with an ROC AUC of 0.69 for unmet efficacy failures and 0.79 for positive efficacy. No studies have identified or predicted drugs and gene association using GAT.

Our study used GAT and Graph Sample and Aggregation (Graph SAGE) to predict drug-gene associations within OSCC, particularly emphasizing GR gene interactions [18]. GAT employs attention mechanisms to evaluate the importance of adjacent nodes, thereby improving the representation of drug-gene relationships. Meanwhile, Graph SAGE facilitates scalability in extensive biological networks by sampling and aggregating information from a node’s neighborhood. By integrating these computational approaches, our objective was to uncover the interactions between Nuclear Receptor Subfamily 3 Group C Member 1 (NR3C1) and specific genes, thus contributing to the understanding of therapeutic responses and aiding in developing personalized treatment strategies for OSCC by predicting drug-gene interaction.

## Methods

### Dataset preparation

**Fig 1:**
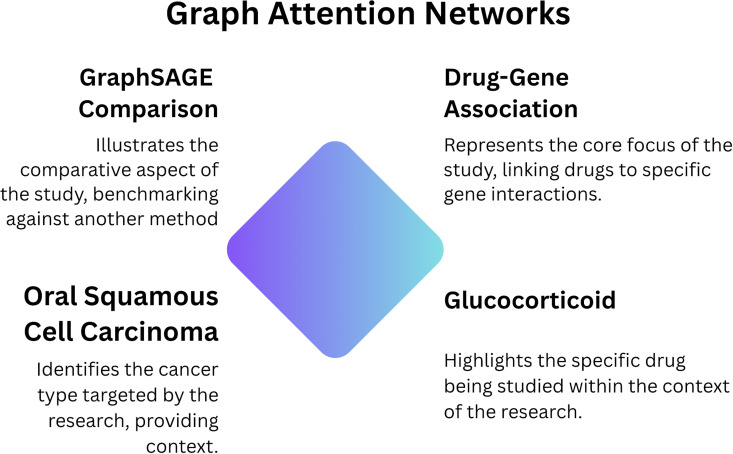
Schema of this study.

Using the probe drugs tool [19], we retrieved drugs and genes associated with glucocorticoid receptors, and this data consists of id, gene name, target name, and biochemical activity. Using Cytoscape, an interactome was created, and with the cytohubba plugin, top hub drugs and genes were identified using the maximum clique centrality method (**Fig 1**).

This dataset consists of 415 drugs and genes with their activity and with nodes as drugs and genes and edges as target_name, edge weight as activity score, and all others as node features.data were subjected to 80 percent training and 20 percent test and subjected to graph attention and graph SAGE. The dataset compiles 415 drug-gene pairs with activity scores, biochemical features, and node-edge specifications from the Probe & Drugs portal. It is well-annotated with biochemical activity and feature-rich nodes, allowing prioritization of clinically relevant genes/drugs This study compared two graph neural network architectures, GAT and GraphSAGE, for node classification on a biological network. The network was constructed using gene names as nodes, with target types as edges and biochemical activity as edge weights. The input features for each node were derived from the encoded representations of the gene names and their associated compound names as node features.

For the GAT model, we implemented a sophisticated two-layer architecture. The first layer comprised 8 multi-head attention mechanisms, each with 16 hidden units, succeeded by an Exponential Linear Unit (ELU) activation function to introduce non-linearity. A dropout regularization technique with a rate of 0.6 was employed both pre- and post-activation to mitigate overfitting. The second layer utilized a singular attention head to yield the conclusive output, incorporating a dropout rate of 0.6 to enhance generalizability. The GAT model was optimized using the Adam optimization algorithm with a learning rate of 0.01 and a weight decay of 5e-4, ensuring robust convergence.

Similarly, the GraphSAGE model also featured a dual-layer architecture. Both layers implemented the mean aggregator function to proficiently assimilate information from proximal neighboring nodes. The initial layer transformed the input feature set into a 16-dimensional latent space representation, followed by a Rectified Linear Unit (ReLU) activation function to induce sparsity. A dropout rate of 0.5 was applied post-activation to further prevent overfitting. The second layer subsequently generated the final output representation. GraphSAGE was trained utilizing the Adam optimizer with a learning rate of 0.01 and a weight decay of 5e-4, analogous to the GAT model. Both models underwent training for 200 epochs on a consistent dataset, partitioned into training (80%) and testing (20%) sets. The negative log-likelihood loss function was adopted as the optimization criterion for both models, ensuring a robust evaluation of model performance.

## Results

**Fig 2:**
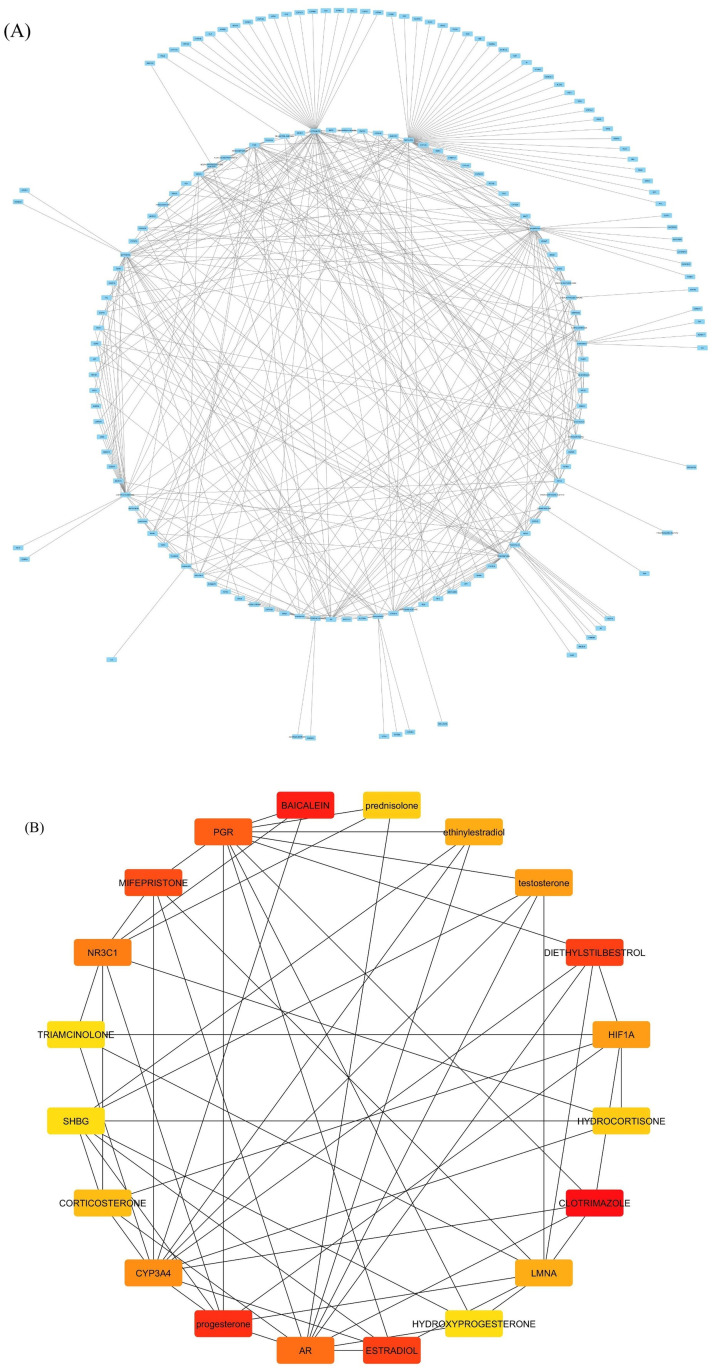
A) Shows the interaction of the drugs and genes associated with glucocorticoid receptors. B) Top hub drugs and genes associated with glucocorticoid receptor.

The network statistics include 174 nodes, 409 edges, an average number of neighbours, a diameter of 6 steps, a radius of 3, and a characteristic path length of 3.14 steps. The clustering coefficient indicates no clustering, and the density indicates a sparse network. The network heterogeneity indicates greater diversity in node connections, while the network centralization indicates moderate centrality. The network has only one connected component, confirming its full connectivity without isolated subgroups. The analysis time is 0.079 seconds, indicating an efficient computation of the network statistics. The network’s low density reflects potential connections that do not exist, and the high heterogeneity suggests some nodes play more significant roles than others. The network is characterized by moderate connectivity with a central structure and low clustering (Fig 2). Top hub drugs and identified including are Clotrimazole, Mifepristone, Baicalein, NR3C1, Estradiol, Progesterone, Prednisolone, PGR, NPL3C1, CYP3A4, Diethylstilbestroll.

### Graph sage and graph attention results

After training, both models showed high accuracy in classifying the nodes but with notable differences in other performance metrics. The GAT model achieved an accuracy of 0.947 and a macro-averaged F1 score of 0.195. Its macro-averaged Area Under the Receiver Operating Characteristic Curve (AUC-ROC) was 0.514, indicating performance only slightly better than random chance across all classes. The GraphSAGE model, on the other hand, demonstrated superior performance across all metrics. It achieved a slightly higher accuracy of 0.949 but showed a significantly better macro-averaged F1 score of 0.275. Most notably, GraphSAGE’s macro-averaged AUC-ROC was 0.780, substantially outperforming the GAT model in distinguishing between classes. The precision-recall curve, plotted for both models using macro-averaging, visually confirms GraphSAGE’s superior performance. The curve for GraphSAGE shows a larger area under the curve, indicating better precision and recall trade-offs across all classification thresholds.

These results suggest that for this particular biological network, the GraphSAGE model was more effective at learning and generalizing the underlying patterns in the data. Its better F1 score indicates a more balanced precision and recalls performance across all classes. At the same time, the higher AUC-ROC suggests it has a stronger ability to distinguish between different classes. Despite its high accuracy, the GAT model struggles with class imbalance or distinguishing between certain classes, as indicated by its lower F1 score and AUC-ROC.

It’s worth noting that both models achieved high accuracy, which might indicate a class imbalance in the dataset. In such cases, accuracy alone can be misleading, and the F1 score and AUC-ROC provide more informative measures of the models’ performance. The superior performance of GraphSAGE might be attributed to its effective use of the mean aggregator function, which could be particularly well-suited to capturing the relevant neighborhood information in this biological network.

## Discussion

OSCC remains a significant global health issue because of its increasing incidence and associated morbidity and mortality [20]. OSCC is significantly affected by various genetic and molecular determinants, particularly those linked to glucocorticoids and sex hormones, highlighting the intricate interplay between hormonal regulation and oncogenesis [12,13]. The role of glucocorticoids in OSCC is notable as these steroids, produced by the adrenal glands, can modulate the immune response and inflammation, potentially affecting tumor growth and progression. Similarly, sex hormones such as estrogen and androgen are also involved in the pathophysiology of OSCC, as they can influence cellular proliferation and differentiation pathways. Studies in OSCC continue exploring the genetic alterations and molecular pathways that drive OSCC, aiming to develop targeted therapies that can improve patient outcomes [21–23]. Early detection and prevention strategies and understanding the hormonal factors involved are crucial in combating the rising burden of OSCC globally.

In this study, we identified top hub drugs and genes associated with glucocorticoids are Clotrimazole, Mifepristone, Baicalein, NR3C1, Estradiol, Progesterone, Prednisolone, PGR, NPL3C1, CYP3A4, Diethylstilbesterol. The glucocorticoid response is strongly influenced by the expression of key genes such as CYP3A4, NR3C1, and vice-versa, which play vital roles in the body’s hormonal landscape [24–26] Of these, CYP3A4, recognized as one of the most important phase I metabolic enzymes, is pivotal in the biotransformation of a diverse array of endogenous and exogenous substrates, including glucocorticoids [27]. Variability in the expression and activity of this gene can lead to considerable alterations in glucocorticoid bioavailability, consequently modulating tumor dynamics and influencing the efficacy of therapeutic interventions [28]. Meanwhile, the NR3C1 gene, which encodes the glucocorticoid receptor, acts as a critical mediator of cellular responses to stress and inflammation—two processes intimately involved in cancer progression [29]. This receptor facilitates the transcription of target genes that can promote cell survival or apoptosis, depending on the context. Dysregulation of these pathways, genetic variations, epigenetic modifications, or environmental factors can generate an altered tumor microenvironment. This transformed environment may foster OSCC development through various intricate mechanisms, including enhanced cellular proliferation, promoting angiogenesis to secure blood supply, and facilitating immune evasion to escape host defenses [30].

The impact of sex hormones on OSCC is of notable significance within the realm of cancer research [31]. The Androgen Receptor (AR) and Sex Hormone-Binding Globulin (SHBG) have surfaced as pivotal elements in modulating androgenic and estrogenic effects pertinent to oral carcinogenesis [32]. Elevated levels of androgens may contribute to the aggressiveness of OSCC, corroborated by studies linking heightened AR expression to advanced tumor stages [33,34]. In contrast, SHBG serves by sequestering sex hormones, potentially mitigating their biological influence, thus establishing a complex hormonal signaling equilibrium that could influence OSCC risk and prognosis.

Another critical aspect of OSCC pathophysiology is hypoxia, commonly observed within the tumor microenvironment [35]. The hypoxia-inducible factor 1-alpha (HIF1A) gene enables cellular adaptation to hypoxic conditions and propels OSCC progression by activating pathways like angiogenesis and metabolic reprogramming [35]. Tumors with pronounced HIF1A expression typically demonstrate increased aggressiveness and resistance to therapy, emphasizing the importance of targeting hypoxia-associated signaling pathways [36,37]. The Lamin A/C gene (LMNA) maintains nuclear integrity and facilitates signaling pathways that influence cellular functions [38]. Genetic alterations or dysregulated LMNA expression can lead to genomic instability, a hallmark of numerous cancer phenotypes, including OSCC [39].

The Progesterone Receptor (PGR) also significantly impacts cellular proliferation driven by Progesterone. Its presence in OSCC suggests a role in tumor initiation and progression, underscoring the importance of hormonal factors in OSCC [40]. From a pharmacological perspective, the use of glucocorticoids such as triamcinolone, prednisolone, and hydrocortisone represents the duality of these agents. While they function as effective anti-inflammatory drugs, alleviating cancer-related symptoms, they may inadvertently augment tumor proliferation under specific circumstances. The complex and multifaceted effects of glucocorticoids on OSCC necessitate a careful and measured therapeutic approach.

**Fig 3:**
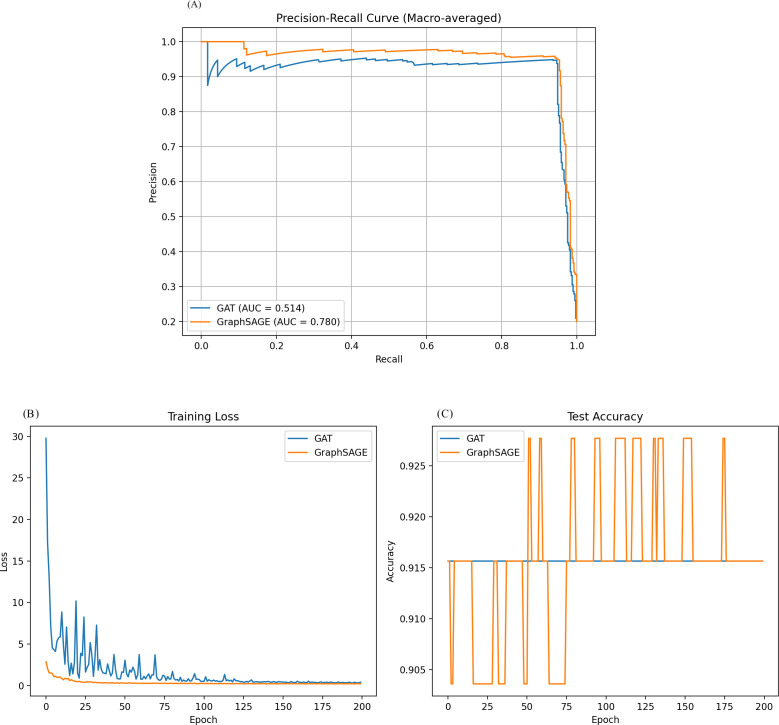
A) Precision-recall curve of the model and B) and C) Epoch loss curve of the model.

In this study, both models showed high accuracy in classifying nodes post-training, with the GAT model slightly better than random chance across all classes (Fig 3). GraphSAGE, on the other hand, outperformed the GAT model in all metrics, with higher accuracy and better macro-averaged F1 score, which is similar to one previous study in predicting drugs using repurposable drugs using a drug repurposing pipeline that integrates SARS-CoV-2 and drug interactions, deep graph neural networks, and in-vitro/population-based validations. The study highlights the top 22 drugs, including Azithromycin, Atorvastatin, Aspirin, Acetaminophen, and Albuterol, and identifies drug combinations that may synergistically target specific COVID-19 [41].

The GAT and GraphSAGE models have shown promising performance in various applications, but there is room for improvement in hybrid architectures that integrate aspects of both models. Hyperparameter tuning could improve model performance, such as grid search, random search, or Bayesian optimization. Expanding to additional datasets and exploring advanced evaluation metrics, such as the Matthews correlation coefficient or AUC-PR, could provide deeper insights into model performance. Layer-wise Relevance Propagation or SHAP can enhance interpretability and explainability. Lastly, incorporating temporal dynamics could improve model capabilities in diverse scenarios. However, both models face limitations, such as performance issues on minority classes, overfitting concerns, computational complexity, and the assumption of homogeneity in node features and relationships [42].

Furthermore, synthetic hormones such as progesterone, ethinylestradiol, and estradiol illustrate the intricate nature of hormonal signaling pathways in OSCC. These compounds may exhibit tumor-promoting and tumor-suppressive activities, contingent on the tumor microenvironment. For example, estrogens may stimulate the proliferation of certain cancer cell phenotypes while promoting apoptosis in others [40]. This paradox highlights the critical role of context in therapeutic decision-making. Agents such as mifepristone, a progesterone receptor antagonist, and clotrimazole, known primarily for its antifungal properties but also affecting steroid biosynthesis, present promising therapeutic avenues targeting hormonal pathways in OSCC [43,44]. A comprehensive understanding of these hormones and pharmacological agents’ precise roles is crucial for developing innovative treatment strategies to overcome the challenges inherent in managing OSCC.

## Conclusion

The complex interrelationship among genetic constituents, hormonal signaling, and pharmacological interventions reveals a convoluted network governing OSCC development and progression through glucocorticoid receptors. By advancing our understanding of these interactions, we can refine therapeutic approaches, paving the way for personalized and more effective treatment regimens for patients confronting this challenging malignancy. Appreciating this complexity may improve survival outcomes and quality of life for individuals impacted by OSCC. The study compared GAT and GraphSAGE in predicting drug-gene associations in oral cancer. GraphSAGE was more effective in accurately predicting these associations due to its ability to capture complex interactions between glucocorticoids and genomic targets. Despite its innovative attention mechanisms, GAT did not match GraphSAGE’s efficacy in this application. Future research should focus on optimizing GraphSAGE and integrating biological data for improved therapeutic strategies.

## Supporting information

S1 File Raw input data used for Graph Attention Network (GAT) modeling of drug-gene associations. (ZIP)
